# Donor-Derived Ehrlichiosis Caused by *Ehrlichia chaffeensis* from Living Donor Kidney Transplant

**DOI:** 10.3201/eid3103.241723

**Published:** 2025-03

**Authors:** Michael J. Scolarici, Daniel Kuehler, Rebecca Osborn, Annie Doyle, Elizabeth K. Schiffman, Alex Garvin, Julian A. Villalba, Carmen J. Ramos, Christopher D. Paddock, Pallavi D. Annambhotla, Marissa Taylor, Johanna S. Salzer, Christopher Saddler, Carrie Thiessen, Raja Kandaswamy, Jon Odorico

**Affiliations:** University of Wisconsin–Madison School of Medicine and Public Health, Madison, Wisconsin, USA (M.J. Scolarici, C. Saddler, C. Thiessen, J. Odorico); University of Minnesota, Minneapolis, Minnesota, USA (D. Kuehler, A. Doyle, R. Kandaswamy); Wisconsin Department of Health Services, Madison (R. Osborn); Minnesota Department of Health, St. Paul, Minnesota, USA (E.K. Schiffman, A. Garvin); Centers for Disease Control and Prevention, Atlanta, Georgia, USA (J.A. Villalba, C.J. Ramos, C.D. Paddock, P.D. Annambhotla, M. Taylor, J.S. Salzer)

**Keywords:** vector-borne infections, bacteria, donor-derived infection, Ehrlichia chaffeensis, living donor, solid organ transplant, Minnesota, Wisconsin, United States

## Abstract

Tickborne infections are challenging to diagnose, particularly among solid organ transplant recipients. We report a US case of donor-derived ehrlichiosis from a living kidney donation that highlights how screening for living donors may miss tickborne infections. Clinicians should consider the epidemiology of the donor when screening donations and evaluating recipients for donor-derived infection.

*Ehrlichia chaffeensis* ehrlichiosis is a tickborne bacterial infection transmitted by the lone star tick (*Amblyomma americanum*) endemic to the southeastern and south-central United States (*1*). Because organ transplant–associated transmission is rare, screening of organ donors for ehrlichiosis is not routine, but infection in transplant recipients is possible (*2**–**5*). We report a case of donor-derived *E. chaffeensis* ehrlichiosis in a Wisconsin, USA, resident from a living kidney donor in Minnesota, USA.

## The Study

The living donor was a 33-year-old man with obesity and unremarkable preoperative examination and serologies who underwent laparoscopic hand-assisted right nephrectomy for National Kidney Registry living unrelated kidney transplant donation at the University of Minnesota (Minneapolis, MN, USA) in June 2023. The donor had an uncomplicated nephrectomy; the total operative time was 4 hours and 29 minutes. On postoperative day (POD) 0, a new erythematous rash on the left hip and lower flank with accompanying myalgia and weakness developed on the donor. His urine output substantially decreased and became cola colored. Creatine kinase (CK) level was 41,155 U/L (reference range 30–200 U/L). Rhabdomyolysis was diagnosed, and the patient received aggressive fluid replacement. Urine myoglobin and CK levels steadily improved, and the donor was discharged on POD 6.

The recipient was a 24-year-old man with end-stage kidney disease secondary to IgA nephropathy who was on peritoneal dialysis. He underwent a National Kidney Registry transplant from the described unrelated donor to the right iliac fossa with antithymocyte globulin induction and peritoneal catheter removal at the University of Wisconsin (Madison, WI, USA). He had an uncomplicated postoperative recovery and was discharged to home on POD 3 with a creatinine level of 1.8 mg/dL (reference range 0.73–1.18 mg/dL) and maintenance immunosuppression (mycophenolate mofetil, tacrolimus, prednisone) and antimicrobial prophylaxis (valganciclovir, trimethoprim/sulfamethoxazole).

A week later, the recipient was readmitted with a fever of 100.7°F (38.2°C), generalized malaise, joint pain, and a perinephric fluid collection measuring 4.9 × 5.1 × 3.8 cm with 69 nucleated cells, predominately macrophages. He received empiric intravenous piperacillin/tazobactam and vancomycin for 48 hours that was then discontinued because admission cultures remained negative. Because of persistent fever that increased to 103°F (39.4°C), worsening renal function, and onset of neutropenia and thrombocytopenia, we performed broad-range 16S rRNA gene PCR on the perinephric fluid, serum Lyme PCR, whole-blood *Babesia* spp. PCR, and whole blood *Ehrlichia* and *Anaplasma* spp. PCR. Progressive kidney injury prompted a graft biopsy.

Further patient history revealed no new sexual partners, no international travel, and minimal outdoor exposure before or after transplantation. Neither he nor his dogs had known recent tick exposures.

The whole-blood *E. chaffeensis* PCR result was positive, and the 16S rRNA gene PCR, followed by sequencing of the perinephric fluid collection, detected *E. chaffeensis*. The patient started oral doxycycline with defervescence within 12 hours. His pancytopenia and renal function improved, and he was discharged on hospitalization day 9 with a creatinine level of 3.6 mg/dL. Two weeks after starting doxycycline, his perinephric drain was removed; doxycycline was stopped 7 days later, for a total 21-day course. Six weeks after transplant, he had a creatinine level of 1.1 mg/dL, as well as unremarkable leukocyte and platelet counts.

Ehrlichiosis is a nationally notifiable disease; therefore, the recipient’s positive *E. chaffeensis* PCR result was reported through the Wisconsin Electronic Disease Surveillance System, initiating a routine investigation by the Wisconsin Department of Health Services. During the interview, the recipient denied recent travel outside his Wisconsin county of residence, outdoor activities, or tick exposures. His recent organ transplantation triggered a multiagency investigation. The donor, a Minnesota resident, reported the following to the Minnesota Department of Health: outdoor exposure in Minnesota and travel to Kansas to hunt 3 weeks before the transplant. While in Kansas, which is an *E. chaffeensis–*endemic state, the donor removed several ticks crawling on his body and clothing, including 1 attached tick; he then experienced an illness the donor attributed to food poisoning the week before transplant.

Tissue biopsies from the transplanted kidney sent to the Centers for Disease Control and Prevention (CDC) revealed mild-to-moderate acute tubular injury and necrosis (Figure, panels A, C, E). Because of clinical and epidemiologic concerns for transplant-transmitted infection of *E. chaffeensis*, we performed an immunohistochemical assay raised against *E. canis* that is known to cross-react with other *Ehrlichia* species, including *E. chaffeensis* (*6*). The immunohistochemical assay highlighted antigens of *Ehrlichia* spp. in intracellular morulae located within circulating and interstitial mononuclear inflammatory cells and endothelial cells of periglomerular capillaries (Figure, panels B, D, F). IgG against *E. chaffeensis* was detected in archived predonation donor serum samples at a titer of 1:128, increasing 4-fold to 1:512 in postdonation donor serum collected on POD 119. The recipient’s pretransplant serum samples were all PCR negative for *E. chaffeensis* (Table).

**Figure:**
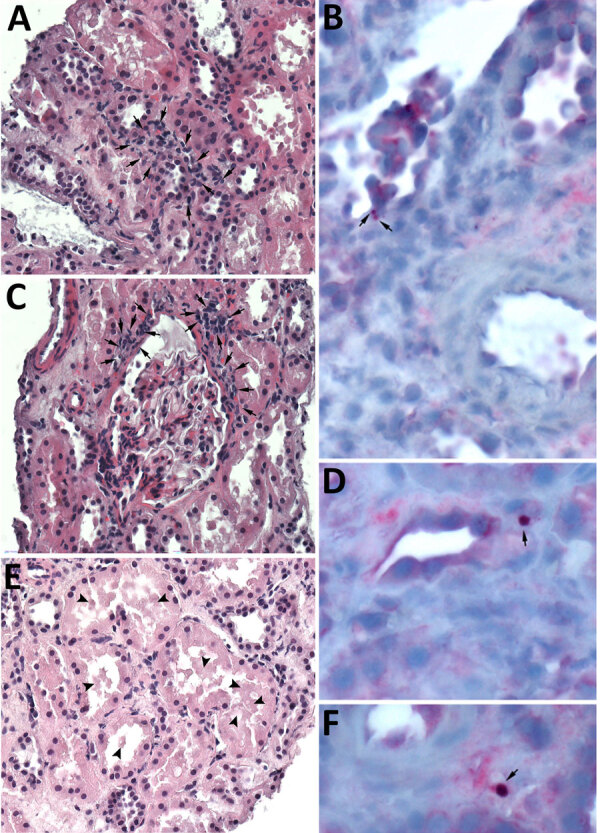
Histopathologic and immunohistochemical staining of renal graft biopsy from donor-derived ehrlichiosis caused by *Ehrlichia chaffeensis* from living donor kidney transplant. We performed an immunohistochemical (IHC) assay using an immunoperoxidase technique, with naphthol fast-red substrate, light hematoxylin counterstaining, and an antibody raised against *E. canis* but known to cross-react with other *Ehrlichia* species, including *E. chaffeensis* and *E. ewingii*, among others. A) Interstitial peritubular mononuclear cell infiltrate (arrows). Hematoxylin and eosin (H&E) stain; original magnification ×400. B) Immunostaining of bacterial antigens from *Ehrlichia* species within 2 morulae in the cytoplasm of reactive endothelial cells of a renal venule (arrows). Original magnification ×630. C) Periglomerular focal interstitial mononuclear cell inflammation (arrows). H&E stain; original magnification ×400. D) Magnification of IHC assay of kidney biopsy indicating a granular immunostaining pattern within intracellular morulae (arrows). Original magnification ×1,000. E) Renal tubules displaying some features of acute tubular necrosis including epithelial cells with condensed chromatin and sloughing of cells into lumina. The tubular lumina are filled with sloughed-off necrotic tubular epithelial cells (arrowheads). Some tubules show near complete luminal occlusion. Casts are not identified. H&E stain; original magnification ×630. F) Another intracellular morulae seen on kidney biopsy by IHC assay (arrow). Original magnification ×1,000.

**Table:** Summary of *Ehrlichia* testing of living donor and recipient from donor-derived ehrlichiosis caused by *Ehrlichia chaffeensis* from living donor kidney transplant*

Days from kidney donation	Specimen	IgG IFA titer†	Real-time PCR‡	16S rRNA gene PCR§	Immunohistochemical assay¶
Donor					
−10	Serum	1:128	Negative	ND	ND
0	Serum	1:128	Negative	ND	ND
20	Serum	1:256	ND	ND	ND
119	Serum	1:512	ND	ND	ND
Recipient					
−15	Serum	ND	Negative	ND	ND
0	Serum	ND	Negative	ND	ND
11	Perinephric fluid	ND	ND	*E. chaffeensis* detected	ND
14	Blood	ND	Positive	ND	ND
16	Renal tissue biopsy	ND	ND	ND	*Ehrlichia* spp. antigens detected

*IFA, indirect fluorescent antibody; ND, not done. †Testing performed at the Centers for Disease Control and Prevention. ‡Testing performed at the Centers for Disease Control and Prevention and Mayo Clinic Laboratories. Negative results defined as no bacterial DNA detected by real-time PCR. §Testing performed at University of Wisconsin Hospital Laboratory. ¶Testing performed at the Centers for Disease Control and Prevention.

The laboratory evidence, exposure history, and epidemiology of ehrlichiosis strongly support donor-derived transmission of *E. chaffeensis* initially acquired by the donor through a tick bite in Kansas 3 weeks before donation (*7*). That case highlights the importance of rapid communication between transplant centers when donor-derived infections are suspected and the value of a parallel surveillance system for tickborne infections leading to a comprehensive investigation between 2 state health departments and the assistance of CDC reference laboratories.

Although routine screening of all living donors for laboratory evidence of ehrlichiosis is not justified, this case study emphasizes the importance of asking living donors and deceased donor next of kin about recent travel and tick exposures, given the perioperative risk to both living donors and recipients. Donor-derived infection from a living donor is unique and is definitive evidence that acute infection with *Ehrlichia* spp. preoperatively developed in the donor. A prior report showed posttransplant ehrlichiosis in 2 kidney recipients with no exposure to ticks transmitted from a deceased donor with increased risk for tickborne disease but no positive donor testing (*5*). Another study highlighted 2 clusters of donor-derived ehrlichiosis from 2 deceased donors found to have attached ticks on postmortem exam; 1 donor had perimortem serum IgG positive for *E. chaffeensis* but no convalescent titers (*8*).

This recipient’s perinephric fluid 16S rRNA gene PCR was sent for evaluation for typical etiologies of surgical site infection, but it detected *E. chaffeensis*. We cannot determine whether that evaluation represents infected fluid or contamination from the recipient’s circulating infected mononuclear cells. However, that evaluation prompted extended therapy beyond the CDC recommendation of >3 days of doxycycline after defervescence and until clinical improvement (typically a minimum of 5–7 days) (*9*).

Previously published cases have suggested that trimethoprim/sulfamethoxazole use may increase the severity of ehrlichiosis, although an analysis in 2020 did not find a major association between trimethoprim/sulfamethoxazole use and need for intensive care after controlling for underlying conditions and doxycycline treatment delay (*10*). In addition, this case highlights rhabdomyolysis, a relatively uncommon and serious complication of ehrlichiosis (*11*,*12*). This donation surgery was not prolonged, and the CK level was higher than expected for routine postoperative rhabdomyolysis. Recognition of a disproportionately high CK level should trigger further evaluation for a secondary cause such as infection.

## Conclusions

In summary, clinicians should remain vigilant for tickborne infections in potential organ donors, particularly those with known exposure to common disease vectors. Clinicians should maintain a broad differential when evaluating solid organ recipients with febrile syndrome shortly after transplantation, even if the donor, recipient, or both reside in areas where potential pathogens are not endemic.
